# The efficacy of a blended motivational interviewing and problem solving therapy intervention to reduce substance use among patients presenting for emergency services in South Africa: A randomized controlled trial

**DOI:** 10.1186/s13011-015-0042-1

**Published:** 2015-11-14

**Authors:** K. Sorsdahl, D. J. Stein, J. Corrigall, P. Cuijpers, N. Smits, T. Naledi, B. Myers

**Affiliations:** Alan J. Flisher Centre for Public Mental Health, Department of Psychiatry & Mental Health, University of Cape Town, Groote Schuur Hospital, Cape Town, 7925 South Africa; Department of Psychiatry & Mental Health, University of Cape Town, Groote Schuur Hospital, Cape Town, 7925 South Africa; Western Cape Department of Health, 8 Riebeeck Street, Cape Town, 8001 South Africa; Department of Clinical Psychology, Faculty of Psychology and Education, VU University Amsterdam, Amsterdam, The Netherlands; Alcohol, Tobacco, and Other Drug Research Unit, South African Medical Research Council, PO Box 19070, Tygerberg, 7505 South Africa

**Keywords:** Substance use, Problem solving therapy, Emergency departments, Brief intervention

## Abstract

**Background:**

The treatment of substance use disorders is a public health priority, particularly in South Africa where the prevalence of these disorders is high. We tested two peer-counsellor delivered brief interventions (BIs) for risky substance use among adults presenting to emergency departments (EDs) in South Africa.

**Methods:**

In this randomised controlled trial, we enrolled patients presenting to one of three 24-hour EDs who screened at risk for substance use according to the Alcohol, Smoking, and Substance Involvement Screening Test (ASSIST). Eligible patients were randomly allocated to one of three conditions: Motivational Interviewing (MI), blended MI and Problem Solving Therapy (MI-PST) or a Psycho-educational Control Group (CG). The primary outcome was reduction in ASSIST scores at three months follow-up.

**Results:**

Of the 2736 patients screened, 335 met inclusion criteria, were willing to participate in the intervention and were randomised to one of three conditions: 113 to MI, 112 to MI-PST and 110 to CG. ASSIST scores at three months were lower in the MI-PST group than they were in the MI and CG groups (adjusted mean difference of −1.72, 95 % CI −3.36 - -0.08). We recorded no significant difference in ASSIST scores between the CG and MI group (adjusted mean difference of −0.02, 95 % CI −2.01 - 1.96).

**Conclusion:**

With the addition of minimal resources, BIs are feasible to conduct in EDs in a low resourced country. These preliminary findings report that MI-PST appears to be an effective BI for reducing substance use among at risk participants. Further research is required to replicate these findings with effort to limit attrition, to determine whether reductions in substance use are persistent at 6 and 12 month follow-up and whether parallel changes occur in other indications of treatment outcomes, such as injury rates and ED presentations.

**Trial registration:**

This trial registered with the Pan African Clinical Trial Registry (PACTR201308000591418)

## Background

Substance use disorders represent a major public health problem, both globally and in South Africa [1]. These disorders, particularly hazardous and harmful alcohol use, are highly prevalent in the Western Cape Province of South Africa [1, 2]. Problematic substance use is strongly associated with risk for interpersonal violence and injury [3–6], with the prevalence of substance use amongst patients presenting to emergency departments (EDs) being high [7]. In previous South African studies, between 33.0 % and 78.9 % of patients presenting with recent injuries at EDs tested positive for recent alcohol or other drug use [6, 8]. These findings highlight the need for brief interventions (BI) that reduce the risk of further substance-related injuries among these at-risk patients [9].

In most instances, BIs involve providing structured information about substance use, giving advice to change, and developing a personal plan to reduce consumption, usually delivered within a motivational interviewing (MI) framework [10]. Although many studies have found that BIs that address substance use in primary care facilities have positive outcomes [11, 12], these findings have not been replicated in low and middle income countries (LMICs) such as South Africa [13, 14] or consistently replicated in ED settings in high income countries [15–21]. Increasingly MI is being blended with other evidence-based treatments, such as cognitive behavioural therapy (CBT), to enhance intrinsic motivation for behavioural change [22] and to provide the necessary skills to enable change. One brief cognitive-behavioural intervention that shows promise for addressing substance use is Problem Solving Therapy (PST). Evidence suggests that PST is effective for treating common mental disorders [23] in a broad range of cultural settings [24–26]. Recently, a phase one study that aimed to assess the feasibility, acceptability and substance use outcomes of a blended MI-PST intervention among 15 patients presenting to emergency room settings in Cape Town, South Africa found that MI-PST was associated with significant reductions in substance use involvement at the three month follow-up [27].

The aim of this paper is to present findings from a phase two trial of Project STRIVE (Substance use and Trauma InterVention), designed to test the effectiveness of a MI-PST intervention for reducing substance use and preventing substance-related injuries among patients attending EDs in Cape Town, South Africa. The purpose of this trial was to determine whether the additional investment in a blended MI-PST intervention over a brief MI intervention or simple alcohol screening and psycho-education would lead to reductions in alcohol consumption and improve quality of life. We hypothesised that the MI-PST intervention would be more effective for reducing substance use and associated harms compared to an MI intervention and Control Group (CG), and that the MI group would report less substance use than the CG.

## Methods

### Participants

Participants were recruited from one of three EDs located in Cape Town. Patients were eligible if they were ≥ 18 years of age and if they were at moderate to high risk for substance use problems, as measured by the Alcohol, Smoking and Substance Involvement Screening Test [28]. Exclusion criteria included a severely altered mental status, being physically incapable of participating due to severe illness, and being without any detailed locator information. A power calculation indicated that a sample size of 240 would detect a medium effect size (Cohen’s d = 0.5, with power (1—β) set at 0.80 and α = 05).

### Procedure

At each participating ED, peer counsellors approached patients for screening after they were triaged for injury or illness severity and while they waited to be seen by the doctor. Patients were asked to provide written consent for eligibility screening. Patients were screened and recruited at varying times during the day and during at least one 12-hour night shift on the weekend (7 pm–7 am) in order to reflect the busiest periods of the selected EDs. Low risk substance users who were not eligible for the intervention were thanked for their time and encouraged to maintain low risk usage. Eligible moderate and high risk users were asked to provide consent to participate in a substance use risk reduction programme. Patients who provided written consent were enrolled in the study and asked to provide locator information prior to completing an interviewer-administered baseline questionnaire which included questions on substance use, injury and other health risks that took approximately 45 min to complete. After this assessment, participants were randomly assigned to one of three conditions. Participants in the control arm were provided with a brochure on substance-related risks and participants in the MI arm and the MI-PST arm received their allotted intervention sessions. The baseline assessment was re-administered three months after the initial assessment for the control and MI group and 3 months following the final intervention session for the PST group. Participants were given a grocery store voucher valued at ZAR 30 (about USD 4) for completion of each assessment. Ethical approval for this study was provided by the Research Ethics Committee from the University of Cape Town’s Faculty of Health Science. We initiated participant recruitment in March 2012 and finished all follow-ups in March 2013. This trial registered with the Pan African Clinical Trial Registry (PACTR201308000591418).

### Randomisation and masking

Treatment allocation was by numbered sealed, opaque envelopes, which were generated by random number tables by a research worker not involved in the delivery of the intervention. In order to limit a socially desirable response set, peer counsellors who delivered the interventions did not conduct the follow-up assessment and interviewers were blinded to the treatment allocation.

### Interventions

#### MI arm

An ASSIST-Linked BI, developed by the World Health Organisation, was provided in this condition [29]. The duration of this brief intervention was approximately 20 min.

#### MI-PST arm

The five session MI-PST intervention was adapted from an intervention previously tested among South Africans experiencing psychological distress in disadvantaged communities [25]. The first session was the ASSIST-linked BI which occurred directly after completion of the baseline assessment. After completing the first session, participants then returned to the community health centre where the ED is located for four further sessions that focused on developing and practicing skills to address life problems. These sessions were spaced approximately one week apart and were between 45 and 60 min in duration (Table 1). During these sessions, the counsellor and the participant collaborated to identify problems occurring in the participant’s life, and focused on exploring one or more of these problems while the counsellor taught the participant a structured PST approach to addressing problems. The participant was required to complete homework for each session; providing an opportunity for participants to apply the skills they acquired during their sessions.

**Table 1:** Summary of blended MI and PST sessions and objectives

At baseline	· Conduct screening/assessment of alcohol use
30 min	· Provide feedback on results of screening/assessment
	· Increase knowledge of how alcohol use impacts on physical and mental health
	· Use MI to build rapport and develop readiness to change
	° Assess readiness to change (using readiness ruler for confidence and importance)
	° Assess pros and cons of change (Decision-balance exercise)
	° Use MI to try and shift participant and elicit a commitment to change
Session 1	Patient check-in (using MI) (*5 min*)
60 minutes	· Build the Rationale for PST (15 min):
	· Explain the structure of PST
	· Explain the link between problems and alcohol use, and the rationale for PST
	· Establish positive problem orientation
	· Describe the steps of PST
	· Build the rationale for activity scheduling
	· Describe the steps of Problem Solving (15 min)
	· First Problem Solving Session with counsellor (using the steps) and describe homework (25 min)
Session 2	· Patient check-in (using MI) (5 min)
40 minutes	· Review homework from previous week and challenges/difficulties (5 min)
	° Elicit positive change talk and affirm commitment to change using MI techniques
	° Review PST steps and affirm attempts to change
	· Explain what can be done about problems that are not important (coping with negative thoughts) (10 min)
	· Second Problem Solving Session with counsellor and an exercise (25 min)
Session 3	· Review practice exercises from session 3 (5 min)
40 minutes	· Explain what can be done about problems that are important but cannot be solved (5 min)
	· Third Problem Solving Session with counsellor and an exercise (30 min)
Session 4	· Review practice exercises from session 3 (5 min)
40 minutes	· Fourth Problem Solving Session with counsellor and an exercise (30 min)
	· Use MI to affirm progress to date and discuss way forward and follow-sup (5 min)

#### Control arm

Participants in this study arm were provided with a brochure providing information on the effects of substance use. No additional counselling was provided.

### Health counsellor training and fidelity

The five counsellors who conducted the brief intervention had a bachelors-level education or equivalent experience and originated from the communities served by the selected emergency services. These peer counsellors received 18 h of training in MI provided by a MI-certified trainer which included proficiency testing. They also received three half-day booster trainings to limit intervention drift and ensure that the MI skills were being applied appropriately. These peer counsellors also completed 12 h of training in PST that also included proficiency testing. In addition to the intervention training, peer counsellors received training in (i) substance use and the risks associated with substance use, (ii) using and scoring the ASSIST, (iii) ethics of research and importance of maintaining confidentiality and reporting adverse events, (iv) the intervention protocol, and (v) the process of referring patients for specialised care. To ensure intervention fidelity, counsellors had a checklist to ensure all aspects of the intervention were provided. They also participated in biweekly supervision and debriefing sessions.

### Outcome measures

#### Primary outcome

The ASSIST was the primary outcome measure for this study. The ASSIST categorises people into low, moderate, or high risk for substance-related problems [28]. Low risk indicates that the participant is at low risk for problems from their current pattern of substance use (with scores of 0–10 for alcohol and <4 for drugs). Moderate risk indicates that the person is at risk for substance-related problems, with scores of 11–26 for alcohol and 4–26 for drugs. Scores >26 indicate that the participant is at high risk of experiencing severe substance-related problems.

#### Secondary outcomes

These included: the Center for Epidemiological Studies Depression Scale (CES-D) and frequency of substance-related injury, physical and verbal violence, and police interaction. Given the high levels of mental health comorbidity in South Africa, particularly among alcohol-using populations [1], and as unaddressed depression could lead to poor responses to alcohol reduction interventions, we included depression as a secondary outcome. The CES-D is designed to measure common symptoms of depression in the general population and consists of 20 self-rated items. Each item is rated on a four-point Likert scale, ranging from 0 (indicating no symptom presence) to 3 (indicating the presence of symptoms most of the time). Composite scale scores range from 0 to 60, with a score of 16 or higher signifying clinically meaningful depression [30]. Frequency of substance-related injury was assessed by the following questions: “How many times in the past 3 months were you injured as a result of your substance use?” and “Thinking about the times you were injured, on how many occasions did you seek medical treatment?” Frequency of substance-related violence was determined by the following questions, “How many times in the last 3 months have you got in a physical fight due to your substance use?” and “How many times in the last 3 months have you got in a verbal argument/fight due to your substance use?” The responses for these questions were discrete. Frequency of police interaction was determined by the following question: “In the past three months how many times have you been in trouble with the police?”

### Statistical analysis

Attrition differed by study condition: for the MI-PST condition, dropout occurred during treatment and follow-up; for the MI and CG arms there was no dropout during treatment. We replaced missing data using multiple imputation by chained equations, as described by Royston [31] in SPSS. We incorporated baseline ASSIST and CES-D scores, age, gender, and race in the imputation model to estimate the missing data in the primary and secondary outcomes. Data were imputed 10 times and on each imputed data file, the analyses were performed. The ten sets of outcomes were then pooled to get a single set of results using multiple imputation inference [32]. This approach has significant advantages over single imputation or listwise deletion [33].

Outcome comparisons between intervention arms were addressed by Helmert contrasts under an Ancova model, using baseline scores as a covariate, and treatment condition as a factor. The first contrast compared the adjusted means of MI-PST vs. MI and CG combined; the second contrast compared the adjusted means of MI and CG condition. The same procedure was followed for secondary outcomes.

## Results

### Participant flow

**Fig. 1:**
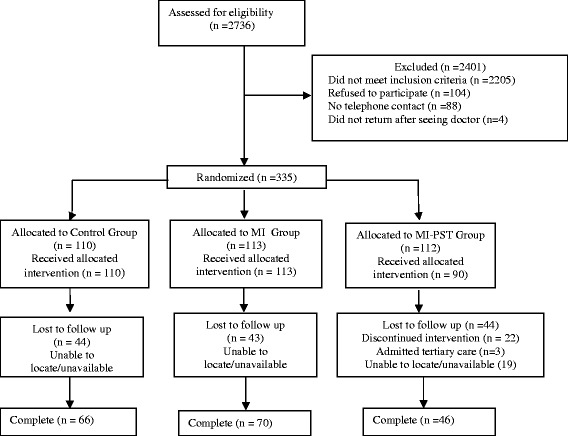
Participant flow chart

A total of 2736 patients presenting for emergency services were screened for possible study inclusion. Of these patients, 19 % (*N* = 531) were at moderate to high risk and met substance use criteria for participation in the intervention study. From these patients, 88 (17 %) were excluded as they were unable to provide locator information. These excluded patients did not differ significantly from those patients who were eligible to participate. Of the remaining 443 eligible patients, 104 (19.6 %) refused to participate mainly because of pain or feeling that they did not have a substance use problem and 4 did not return for their assessment after receiving medical care. These participants had significantly lower scores on the ASSIST (M = 15.68, SD = 7.60) than the 335 participants who were willing to participate (M = 17.45, SD = 9.08). In total, 335 (73.8 %) of the potentially eligible participants were willing to participate in the intervention programme. These participants were randomised to one of three study arms: 113 in the MI, 112 to the MI-PST condition and 110 to the CG. Twelve participants withdrew from the MI-PST group prior to receiving the intervention (Fig. 1).

### Sample description

Table 2 shows the demographic characteristics of each group and the overall sample. More Black ED patients (58 %) participated in the intervention than Coloured (41 %). In this context ‘Black African’ refers to not being ‘Coloured’, white or Indian/Asian. While the term ‘Coloured’ refers to an ethnic group of people who possess some degree of sub-Saharan ancestry, but not enough to be considered Black African during apartheid. These are commonly used markers of race identity in South Africa. The majority of participants were male (*n* = 218; 65.5 %), with an average age of 28 years old (range 18–75). Most participants were single (*n* = 272; 82.9 %) and unemployed (*n* = 184; 55.6 %). Most patients (72.2 %) presented to the ED with an injury, with the remainder seeking treatment for symptoms of ill health (27.8 %). Most patients presenting with an injury were under the influence of substances at the time (*n* = 197; 59.2 %). Alcohol was the most frequently reported substance, with 286 participants (85 %) reporting alcohol use, 24 (7.0 %) participants reporting cannabis use, and 20 (6.0 %) participants reporting methamphetamine use.

**Table 2:** Baseline characteristics of participants

	Total (*N* = 335)	Control (*N* = 110)	MI (*N* = 113)	MI-PST (112)
Age (Mean, range)	28 (18–75)	27 (18–75)	28 (18–65)	28.5 (18–61)
Gender				
Male N (%)	218 (65.5)	68 (62.4)	71 (63.4)	79 (70.5)
Female N (%)	115 (34.5)	41 (37.6)	41 (36.6)	33 (29.5)
Race				
Black N (%)	195 (58.2)	58 (53.7)	68 (60.7)	69 (61.6)
Coloured N (%)	135 (40.7)	49 (45.4)	44 (39.30	42 (37.5)
White/Asian N (%)	2 (0.6)	1 (0.9)	0	1 (0.9)
Marital Status				
Single N (%)	272 (82.9)	91 (85.8)	89 (80.2)	92 (82.9)
Married or attached N (%)	56 (17.1)	14 (14.2)	22 (19.8)	19 (17.1)
Education				
Did not finish high school N (%)	168 (50.1)	58 (52.7)	56 (49.6)	54 (48.2)
Finished high school N (%)	167 (49.9)	52 (47.30	57 (50.4)	58 (51.8)
Employment				
Employed N (%)	147 (44.4)	45 (41.3)	46 (40.7)	56 (51.4)
Unemployed N (%)	184 (55.6)	64 (58.7)	67 (59.3)	53 (48.6)
Presented with Injury				
Yes	242 (72.2)	78 (70.9)	82 (72.6)	82 (73.2)
No	93 (27.8)	32 (29.1)	31 (27.4)	30 (26.8)
Intent of Injury				
Intentional N (%)	185 (76.5)	49 (62.0)	43 (52.4)	46 (56.1)
Non-intentional (N (%)	57 (23.5)	20 (38.0)	39 (47.6)	36 (32.1)
Under the influence when injured				
Yes N (%)	197 (59.2)	70 (63.6)	72 (64.9)	57 (50.9)
No N (%)	136 (40.8)	40 (36.4)	39 (35.1)	55 (49.1)
Substance Use Involvement Score (Total)	19 (6–38)	19 (7–35)	19.75 (6.57)	18 (6–35)
Alcohol (*n* = 286)	19 (10–38)	19 (10–30)	20 (10–38)	17 (10–30)
Dagga (*n* = 24)	20.5 (8–35)	21.5 (12–27)	18.09 (7.9)	19.9 (10.1)
Cocaine (*n* = 1)	26 (26–26)	n/a	n/a	n/a
Methamphetamine (*n* = 20)	23.5 (6–35)	19 (7–35)	27 (17–32)	23.3 (9.98)
Mandrax (*n* = 4)	9.5 (6–23)	23 (23–23)	6 (6–13)	n/a

### Attrition and follow-up rates

Of the 112 patients who were randomised to the MI-PST condition, 46 completed all five sessions of PST. Of those who did not complete the intervention, 22 patients only attended their first appointment and were unable to be located for further appointments or any follow-up sessions, despite repeated attempts. Three participants discontinued the intervention as they were admitted to tertiary care and were unavailable for the follow-up.

A total of 182 (54 %) participants completed the three month follow-up: 70 (62 %) in the MI, 46 (42 %) in the MI-PST and 66 (60 %) in the CG arms. Race (*χ*^*2*^ *=* 9.04, df = 2, *p* = 0.011*)* and treatment condition (*χ*^*2*^ *=* 10.97, df = 2, *p* < 0.001*)* were the only variables that distinguished between participants who completed the three month follow-up and those who did not*.* Coloured respondents were more likely than Black African participants, and participants in the MI arm were more likely than those in the other arms to complete the follow-up assessment.

### Outcomes

While ASSIST scores decreased from baseline to follow-up in all three arms, Helmert contrasts under an Ancova model found that ASSIST scores at three months were significantly lower in the MI-PST group than they were in the MI and CG groups (t (332) = −2.08, *p* = 0.04) with an adjusted mean difference of −1.72. Given the high level of attrition reported in the present study, it is not surprising that a much larger difference in ASSIST scores was reported when the data were analysed for completers only (t (332) = −4.32, *p* > 0.001) with an adjusted mean difference of 4.27. There were no significant differences found between the MI and CG group (t (332) = − 0.02, *p* = 0.98; Table 3).

**Table 3:** Intention to treat- comparison of groups on primary and secondary outcomes

	Control	MI	MI-PST	MI vs Control (Contrast 1)		MI/CG vs MI-PST (Contrast 2)	
	(mean [1])	(mean [1])	(mean [1])	Adjusted Mean	95 % CI	Adjusted Mean	95 % CI
				(diff [SE])		(difference [SE])	
Substance use: ASSIST							
Baseline	19.30 (5.78)	19.96 (6.49)	18.71 (6.32)				
3 month follow-up	11.91 (6.94)	12.28 (6.81)	9.89 (6.64)				
Adjusted Means (SE)	11.93 (0.66)	11.95 (0.72)	10.22 (0.78)	−0.02 (1.00)	−2.01-1.96	−1.72 (0.82)	−3.36- -0.08*
Depression: CES-D							
Baseline	24.56 (6.02)	23.93 (5.43)	27.28 (8.22)				
3 month follow-up	20.13 (7.09)	17.77 (8.12)	16.64 (8.17)				
Adjusted Means (SE)	17.78 (1.30)	15.64 (1.21)	13.38 (1.49)	2.15 (1.59)	−0.99-5.29	−3.33 (1.46)	−6.24- -0.42**
Verbal arguments							
Baseline	0.82 (1.56)	0.59 (1.21)	0.92 (3.02)				
3 month follow-up	0.76 (0.97)	0.23 (0.77)	0.71 (1.18)				
Adjusted Means (SE)	0.60 (0.10)	0.37 (0.10)	0.52 (0.13)	0.24 (0.15)	−0.05-0.54	0.04 (0.15)	−0.27-0.34
Physical fights							
Baseline	0.49 (0.73)	0.50 (0.97)	0.55 (1.41)				
3 month follow-up	0.45 (0.63)	0.28 (0.84)	0.23 (0.71)				
Adjusted Means (SE)	0.36 (0.07)	0.30 (0.07)	0.24 (0.07)	0.06 (0.09)	0.12-0.25	0.04 (0.15)	−0.25-0.07
Police interactions							
Baseline	0.25 (0.63)	0.15 (0.36)	0.55 (1.41)				
3 month follow-up	0.23 (0.60)	0.23 (0.56)	0.16 (0.59)				
Adjusted Means (SE)	0.19 (0.06)	0.24 (0.05)	0.16 (0.05)	−0.05 (0.07)	−0.19-0.09	−0.05 (0.07)	−0.19- -0.09
Injuries							
Baseline	0.76 (0.72)	0.67 (0.84)	0.19 (0.53)				
3 month follow-up	0.23 (0.49)	0.27 (0.53)	0.36 (0.54)				
Adjusted Means (SE)	0.25 (0.06)	0.26 (0.05)	0.30 (0.06)	−0.01 (0.08)	−0.16-0.14	0.04 (0.07)	−0.10-0.18
Healthcare visits							
Baseline	0.67 (0.59)	0.55 (0.60)	0.45 (0.75)				
3 month follow-up	0.54 (0.63)	0.49 (0.84)	0.56 (1.24)				
Adjusted Means (SE)	0.43 (0.09)	0.49 (0.08)	0.61 (0.08)	−0.06 (0.11)	−0.28-0.16	0.15 (0.10)	−0.05-0.35

*(t (332) = −2.08, *p* = 0.04)

**(t (170) = 2.72, *p* = *p* > 0.001)

### Secondary outcomes

When we only considered participants who screened at risk for depression (CES-D score ≥16) using Helmert contrasts under an Ancova model, participants in the MI-PST arm reported significantly lower CES-D scores relative to the combined MI and CG arms at follow-up (t (170) = 2.72, *p* = *p* > 0.001) with an adjusted mean difference of 3.33. There were no significant differences in frequency of substance-related injury, physical or verbal violence or police interaction between the MI-PST arm and the combined MI and CG arm (Table 3).

## Discussion

This study is among the first to examine the effectiveness of a blended MI-PST intervention for substance use within ED settings in a LMIC context. Findings suggest that screening and BI for substance use are feasible to implement in ED settings, and that MI-PST is a potentially effective intervention for reducing risk for substance-related problems and depression among patients presenting for ED services relative to a combined MI and psycho-education control group.

First, findings indicate that it is not only feasible to screen large numbers of people presenting for ED services in Cape Town, South Africa for possible inclusion in a substance use risk reduction intervention, but that such an intervention programme is urgently needed among this population. The need for a substance-related risk reduction intervention was high among the patients screened, with approximately one fifth of patients screened meeting criteria for moderate to high risk substance use. In addition, we found that a high proportion (close to three-quarters) of these eligible patients were willing to participate in a brief intervention to reduce their substance use, thus demonstrating the feasibility of recruiting patients from ED settings to participate in BI for substance use. It is important to note that given the high attrition in the MI-PST group, a reduction in the number of sessions should be considered.

Second, MI-PST may be an efficacious intervention for reducing risk for substance use and depression among patients presenting for ED services. This findings expands current knowledge on the potential value of PST, which to date has focused on the efficacy of PST for addressing mood disorders [26]. Our study is among the first to show that a blended MI-PST intervention may be an effective transdiagnostic treatment that targets shared risk factors for both depression and substance use disorders. Therefore, instead of designing treatments aimed to address one specific diagnosis, given the limited resources in LMICs it may be worth developing treatments that are equally effective at addressing a wider range of mental disorders. However, one of the main limitations of the present study is the high attrition rates for follow-up. In our experience it is feasible to follow-up with substance using populations should sufficient funds be available to conduct follow-ups in the community or provide funding for travel. Furthermore, as our MI-PST intervention was considerably longer than the intervention provided in the MI condition, and because our findings could have arisen from differences in intervention dosage rather than intervention content, this should be viewed as a preliminary finding only. Whether this intervention is considerably more efficacious than MI alone remains to be confirmed through a randomised controlled trial that matches the treatment conditions on dosage and has sufficient systems in place to limit attrition. Nonetheless our findings do provide evidence of the positive effects of an MI-PST intervention on both substance use and depression.

The data reported here also show no differences between the MI and CG arms on outcomes. These findings suggest that screening for substance-related problems may be as effective in prompting short-term reductions in substance use as a one session BI. These results are largely consistent with some previous research [34], and it is not altogether surprising as previous studies conducted in South Africa and have also found that screening for alcohol use is as effective in prompting short-term reductions in hazardous alcohol use as one session BIs that use MI [27]. Together these findings suggest that merely raising the subject of substance use with someone who has just experienced a negative substance-related consequence (such as an injury) may prompt the participant to make changes to their pattern of use. Given our short follow-up period, it is unclear however whether these short-term reductions in substance use associated with screening are sustained over time; future studies should consider utilising longer follow-up periods.

Third, findings suggest that there were no significant differences on the frequency of substance-related injury, physical or verbal violence or police interaction between the MI-PST arm compared to the combined MI and CG arm or the MI and CG arm. This is inconsistent with previous studies conducted among patients attending ED which found that BI diminished the likelihood of future alcohol-related injuries [35]. Our lack of findings could be due to the short follow-up period used in this study; three months may not be long enough to establish the impact of the BIs on future injuries, episodes of violence and frequency of contact with police and healthcare services. Other unmeasured contextual factors (such as community drinking norms, crime and safety in communities) may also explain this lack of findings. Alternatively, perhaps the MI-PST intervention needs to be strengthened to explicitly address other risks for interpersonal violence and injury.

Apart from the aforementioned limitations, there are a few other limitations that must be considered when interpreting study findings. First, the study relied on self-reports of substance use. Although there is evidence available suggesting that self-reports of drinking are generally reliable in EDs [36], the low number of respondents disclosing drug use in the present study is of concern given that the communities we recruited from are known to have high population prevalence rates for cannabis and methamphetamine use; future studies should consider using biological testing to confirm self-reported data on illicit drug use. Second, the results of the present study may not be generalisable to patient groups not represented in the study, such as adolescents. Third, we were unable to contact participants who were randomized to the MI-PST group who did not attend any sessions. All of these participants did not participate in the three month follow-up. This may have implications for future studies of this nature. Finally, clinical trials that match intervention conditions on dosage and that examine outcomes over longer periods of time are needed before definitive statements about the efficacy of MI-PST for reducing substance use risks can be made.

## Conclusion

Despite these limitations, this study provides preliminary evidence that a blended MI-PST intervention is not only feasible to implement among patients presenting for ED services in a LMIC such as South Africa but also has promising outcomes for substance use and depression. While these are two major risk factors for injury, the efficacy of MI-PST for reducing risk of injury is as yet unknown.

## Abbreviations

ASSIST: Alcohol, Smoking, and Substance Involvement Screening Test
BI: Brief intervention
CES-D: Center for Epidemiological Studies Depression Scale
CG: Control Group
ED: emergency departments
LMIC: low and middle income countries
MI: Motivational Interviewing
PST: Problem Solving Therapy
